# The Relationship between *H. pylori* Infection and Osteoporosis in Japan

**DOI:** 10.1155/2014/340765

**Published:** 2014-06-12

**Authors:** Daisuke Asaoka, Akihito Nagahara, Mariko Hojo, Hitoshi Sasaki, Yuji Shimada, Takashi Yoshizawa, Taro Osada, Sumio Watanabe

**Affiliations:** Department of Gastroenterology, School of Medicine, Juntendo University, 2-1-1 Hongo, Bunkyo-ku, Tokyo 113-8421, Japan

## Abstract

*Background and Objective*. *H. pylori* infection causes a chronic inflammation in the gastric mucosa. However, this local inflammation may result in extra-digestive conditions. Our aim is to investigate the relationship between *H. pylori* infection and osteoporosis in Japan. *Methods*. This cross-sectional study was conducted among outpatients at the Juntendo University Hospital between 2008 and 2014. Participants for patient profile, *H. pylori* infection status, comorbidity, internal medical therapies, lumbar dual-energy X-ray absorptiometry (DXA), and bone turnover marker were collected and upper gastrointestinal endoscopy for reflux esophagitis, hiatal hernia, peptic ulcer disease (PUD), and endoscopic gastric mucosal atrophy (EGA) was performed. The diagnosis of osteoporosis was performed in accordance with the Japanese criteria. We investigated risk factors of osteoporosis. *Results*. Of the eligible 200 study subjects, 41 cases were of osteoporosis. Bivariate analysis showed that age, being female, BMI, alcohol, smoking,* H. pylori*, bone-specific ALP, PUD, and EGA were related to osteoporosis. Multivariate analysis showed that age (OR 1.13; 95%CI 1.07–1.20), being female (OR 4.77; 95%CI 1.78–12.77), BMI (OR 0.79; 95%CI 0.68–0.92), *H. pylori* (OR 5.33; 95%CI 1.73–16.42), and PUD (OR 4.98; 95%CI 1.51–16.45) were related to osteoporosis. *Conclusions*. *H. pylori* infection may be a risk factor of osteoporosis in Japan.

## 1. Introduction

*Helicobacter pylori *(*H. pylori*) infection is a leading worldwide infectious disease that affects more than half of the world's population.* H. pylori* is a gram-negative, spiral-shaped pathogenic bacterium that colonizes the gastric epithelium specifically and causes atrophic gastritis, peptic ulcer disease, and gastric malignancies [1, 2].* H. pylori* infection causes a chronic cellular inflammatory response in the gastric mucosa. However, the effects of this local inflammation may not be confined to the digestive tract and may result in extra-digestive conditions [3], such as osteoporosis [4]. On the other hand, the malabsorption of dietary calcium may be a crucial cause of osteoporosis [5].

Osteoporosis is a silent disease characterized by decreased bone density with a risk of spine and hip fracture. Since spine and hip fracture enormously affects life prognosis [6] and social burden [7], World Health Organization (WHO) sets 2000 through 2010 as “The Bone and Joint Decade” [8]. In particular, hip fractures due to osteoporosis are a major factor of bedridden elderly person in aging populations.* H. pylori* infection has been linked epidemiologically to extra-intestinal diseases [9, 10]; however, few studies have examined the relationship between* H. pylori* infection and osteoporosis. Furthermore, all of the studies regarding the relationship between* H. pylori* infection and osteoporosis were performed in Western countries. Geographical differences exist in the genetic polymorphisms of* H. pylori* pathogenicity, such as CagA, which is related to the diversity of disease [11]. In this study, we investigated the relationship between* H. pylori *infection and osteoporosis in Japan.

## 2. Materials and Methods

### 2.1. Patients

This is a cross-sectional study conducted among outpatients (≥50 years old) at our department between March 2008 and February 2014. Participants were examined for age, sex, body mass index (BMI), alcohol consumption and smoking, comorbidity (diabetes mellitus (type 2) and hypertension), internal medical therapies (proton pump inhibitor (PPI) and low dose aspirin (LDA)),* H. pylori* infection status, dual-energy X-ray absorptiometry (DXA), lateral vertebral X-rays, bone turnover markers, and the findings of upper gastrointestinal endoscopy.* H. pylori* infection status was assessed by ^13^C-urea breath test (UBT) and/or serum antibodies to* H. pylori*, and we defined a positive result for any of these tests as positive for* H. pylori* infection. BMI was calculated as body weight divided by the square of body height in meters (kg/m^2^). We defined the cases that used usual dose of PPI and LDA more than a half year as PPI and LDA users. This study was conducted in accordance with the Declaration of Helsinki.

### 2.2. Diagnosis of Osteoporosis

The bone mineral density (BMD) of the lumbar vertebrae 2-4 (L2-4) was measured by DXA using a Discovery A (HOLOGIC, Bedford, Massachusetts). The results are in BMD (g/cm^2^) and young adult mean (YAM). We investigate the presence of fragility fractures in chest and lumbar spine by lateral vertebral X-rays. The diagnosis of osteoporosis was performed in accordance with the 2000 version of the Japanese diagnostic criteria by the Japanese Society for Bone and Mineral Research [12]. Osteoporosis was defined as when the lumbar BMD was less than 70% of the YAM even in those without any prevalent fragility fracture. Osteoporosis was also defined as the presence of fragility fractures in any bone in a person with a BMD of less than 80% of the YAM. Bone turnover markers were also investigated, including serum bone-specific alkaline phosphatase (BAP) (U/L) as a biomarker of bone formation and serum collagen type-I cross-linked N-telopeptide (NTX) (nmolBCE/L) as a biomarker of bone resorption.

### 2.3. The Findings of Upper Gastrointestinal Endoscopy

We investigated the findings of upper gastrointestinal endoscopy (reflux esophagitis (RE), peptic ulcer disease (PUD), hiatal hernia, and endoscopic gastric mucosal atrophy (EGA)). We defined RE as grades A, B, C, and D according to the Los Angeles Classification and PUD as gastric and/or duodenal ulcer and ulcer scar. Hiatal hernia was defined as an apparent separation of the esophagogastric junction and diaphragm impression by more than 2 cm at endoscopy. EGA was classified as C-0 (no atrophy), C-1, C-2, C-3, O-1, O-2, and O-3 type by the Kimura-Takemoto classification system [13], which evaluates the location of the endoscopic atrophic border. Overall, EGA was scored as 0 for C-0 type, 1 for C-1 type, 2 for C-2 type, 3 for C-3 type, 4 for O-1 type, 5 for O-2 type, and 6 for O-3 type.

### 2.4. Exclusion Criteria

We excluded the following comorbidities and drugs that affect bone metabolism as secondary osteoporosis. We excluded patients with the following diseases that affect bone metabolism: gastrectomy, inflammatory bowel disease, malignant diseases (gastric cancer, esophageal cancer, colon cancer, lung cancer, pancreas cancer, liver cancer, bile duct cancer, gallbladder cancer, breast cancer, uterus cancer, ovarian cancer, prostate cancer, bladder cancer, malignant lymphoma, leukemia, and multiple myeloma), chronic kidney disease, diabetes mellitus (type 1), hypo/hyperthyroidism, hypo/hyperparathyroid disorder, and rheumatoid arthritis (including collagen disease). We excluded patients administered the following drugs that affect the bone metabolism: current or previous treatment with glucocorticoids, hormone replacement therapy, thyroid/parathyroid drugs, psychotropic drugs, anticonvulsants, selective estrogen receptor modulators (SERMs), vitamin D, calcium, and bisphosphonate. Furthermore, we excluded patients that had successful eradication of* H. pylori*.

### 2.5. Statistical Analysis

We divided the subjects into two groups, osteoporosis and nonosteoporosis, and investigated the risk factors of osteoporosis between the two groups by bivariate and multivariate analysis. Independent variables with a *P* value of less than 0.20 in the bivariate analysis were included in the multivariate logistic regression analysis (in a backward selection method (likelihood ratio)). Odds ratio and 95% confidence interval were also used to identify the presence and strength of association. Standard techniques for model checking, including the model square test, Hosmer-Lemeshow goodness of fit test, Nagelkerke *R* ^2^, and discriminant hitting ratio were performed to determine the adequacy of the multivariate logistic regression model.

All statistical analyses were performed using SPSS version 19 statistical package. Statistical significance was inferred at *P* values <0.05.

## 3. Results

### 3.1. Patient Profile

Of 275 cases that participated in the study, 75 cases were excluded by the exclusion criteria (64 cases after successful* H. pylori* eradication, 6 cases for malignant disease, 3 cases for hyperthyroidism, and 2 cases for previous treatment with glucocorticoids). The clinical characteristics of the 200 eligible cases are summarized in Table 1.

These cases (95 men (47.5%) and 105 women (52.5%); age range: 50–88 years; mean age: 63.1 ± 8.8 years; mean BMI: 22.9 ± 3.4 kg/m^2^) completed the* H. pylori* infection status, upper gastrointestinal endoscopy, DXA, vertebral X-rays, and bone turnover marker examination. A total of 78 (39.0%) cases consumed alcohol and 62 (31.0%) cases smoked.* H. pylori* positive and* H. pylori* negative cases were 83 (41.5%) and 117 (58.5%) cases, respectively. Diabetes mellitus (type 2) and hypertension cases were 20 (10.0%) and 58 (29.0%) cases, respectively. PPI users and LDA users were 58 (29.0%) and 22 (11.0%) cases, respectively. In male participants (mean age, 62.8 ± 7.7 years; mean BMI, 23.6 ± 2.6 kg/m^2^), a total of 59 (62.1%) cases consumed alcohol and 51 (53.7%) cases smoked.* H. pylori* positive and* H. pylori* negative cases were 38 (40.0%) and 57 (60.0%) cases, respectively. Diabetes mellitus (type 2) and hypertension cases were 12 (12.6%) and 34 (35.8%) cases, respectively. PPI users and LDA users were 32 (33.7%) and 13 (13.7%) cases, respectively. In female participants (mean age, 63.4 ± 9.6 years; mean BMI, 22.2 ± 3.8 kg/m^2^), a total of 19 (18.1%) cases consumed alcohol and 11 (10.5%) cases smoked.* H. pylori* positive and* H. pylori* negative cases were 45 (42.9%) and 60 (57.1%) cases, respectively. Diabetes mellitus (type 2) and hypertension cases were 8 (7.6%) and 24 (22.9%) cases, respectively. PPI users and LDA users were 26 (24.8%) and 9 (8.6%) cases, respectively. In males and females, BMI, the rate of alcohol consumption, and the rate of smoking were decreased in females than in males.

### 3.2. Bone Metabolism

The mean BMD and YAM were 0.88 ± 0.17 g/cm^2^ and 84.9 ± 15.7%, respectively. In males, the mean BMD and YAM were 0.96 ± 0.14 g/cm^2^ and 91.1 ± 13.5%, respectively. In females, the mean BMD and YAM were 0.81 ± 0.16 g/cm^2^ and 79.3 ± 15.6%, respectively. In males and females, mean BMD and YAM were decreased in females than in males. Of the eligible 200 cases, 41 (20.5%) were osteoporosis (8 men and 33 women) and 159 (79.5%) were nonosteoporosis (87 men and 72 women). Of the 41 osteoporosis cases, 33 (80.5%) were female. There are no patients with fragility fractures in lateral vertebral X-rays in this study. Mean BAP and NTX were 22.5 ± 6.5 U/L and 14.2 ± 5.0 nmolBCE/L, respectively. In males, mean BAP and NTX were 21.4 ± 6.0 U/L and 13.6 ± 4.8 nmolBCE/L, respectively. In females, mean BAP and NTX were 23.4 ± 6.6 U/L and 14.8 ± 5.2 nmolBCE/L, respectively. In males and females, mean BAP was increased in females than in males.

### 3.3. The Findings of Upper Gastrointestinal Endoscopy

The cases of reflux esophagitis for LA-grades A, B, C, and D were 7, 2, 0, and 0, respectively. Hiatal hernia and PUD were observed in 91 (45.5%) and 26 (13.0%) cases, respectively. Mean EGA was 1.8 ± 1.9. The cases of EGA for C-0, C-1, C-2, C-3, O-1, O-2, and O-3 were 66, 51, 18, 12, 27, 19, and 7 cases, respectively. In males, the cases of reflux esophagitis for LA-grade A, B, C, and D were 4, 2, 0, and 0, respectively. Hiatal hernia and PUD were observed in 48 (50.5%) and 12 (12.6%) cases, respectively. Mean EGA was 1.9 ± 1.8. The cases of EGA for C-0, C-1, C-2, C-3, O-1, O-2, and O-3 were 28, 26, 11, 3, 16, 9, and 2 cases, respectively. In females, the cases of reflux esophagitis for LA-grades A, B, C, and D were 3, 0, 0, and 0, respectively. Hiatal hernia and PUD were observed in 43 (41.0%) and 14 (13.3%) cases, respectively. Mean EGA was 1.8 ± 2.0. The cases of EGA for C-0, C-1, C-2, C-3, O-1, O-2, and O-3 were 38, 25, 7, 9, 11, 10, and 5 cases, respectively.

### 3.4. Bivariate Analysis

In the nonosteoporosis and osteoporosis groups, age was 61.7 ± 8.5 versus 68.9 ± 7.3 years, female gender in 45.3% (72/159) versus 80.5% (33/41), BMI was 23.4 ± 3.3 versus 21.0 ± 3.1, alcohol consumption in 42.8% (68/159) versus 26.8% (11/41), smoking in 35.8% (57/159) versus 12.2% (5/41),* H. pylori* positive results in 35.8% (57/159) versus 61.0% (25/41), diabetes mellitus (type 2) in 11.3% (18/159) versus 4.9% (2/41), hypertension in 30.2% (48/159) versus 24.4% (10/41), PPI users in 28.3% (45/159) versus 31.7% (13/41), LDA users in 11.3% (18/159) versus 9.8% (4/41), BAP observed in 22.0 ± 6.5 versus 24.4 ± 5.7, NTX observed in 14.0 ± 4.9 versus 15.0 ± 5.6, reflux esophagitis in 5.0% (8/159) versus 2.4% (1/41), hiatal hernia in 47.8% (76/159) versus 36.6% (15/41), PUD in 9.4% (15/159) versus 26.8% (11/41), and EGA as 1.7 ± 1.8 versus 2.4 ± 2.2, respectively. The significant risk factors of osteoporosis were advanced age (*P* < 0.001), female gender (*P* < 0.001), lower BMI (*P* < 0.001), alcohol consumption (*P* = 0.035), smoking (*P* = 0.006),* H. pylori* positive (*P* = 0.002), BAP (*P* = 0.038), presence of PUD (*P* = 0.005), and EGA (*P* = 0.039). The other factors were not related to osteoporosis (Table 2).

### 3.5. Multivariate Logistic Regression Analysis

Models were adjusted for age, gender, BMI, alcohol consumption, smoking,* H. pylori* infection, BAP, PUD, and EGA. The statistical compatibility in multivariate analysis showed as follows; model square test was *P* < 0.01, Hosmer-Lemeshow goodness of fit test *P* = 0.900, Nagelkerke *R* ^2^ 0.454, and discriminant hitting ratio 86.0%. Multivariate logistic regression analysis showed that advanced age (OR 1.13; 95%CI 1.07–1.20, *P* < 0.001), female gender (OR 4.77; 95%CI 1.78–12.77, *P* = 0.002), lower BMI (OR 0.79; 95%CI 0.68–0.92, *P* = 0.002),* H. pylori* positive (OR 5.33; 95%CI 1.73–16.42, *P* = 0.004), and the presence of PUD (OR 4.98; 95%CI 1.51–16.45, *P* = 0.008) were related to osteoporosis (Table 2).

## 4. Discussion

This cross-sectional study clarified the relationship between* H. pylori* infection and osteoporosis in Japanese cases and investigated the patient's profile,* H. pylori* infection status, internal medical therapies, comorbidity, bone turnover markers, and the findings of upper gastrointestinal endoscopy by multivariate analysis. The prevalence rate of* H. pylori* infection in this study was similar to that of Japanese patients ≥50 years of age [14], and the statistical compatibility (model square test < 0.01, Hosmer-Lemeshow goodness of fit test: *P* = 0.900, Nagelkerke *R* ^2^: 0.454, and discriminant hitting ratio: 86.0%) was considered good.* H. pylori* infection is a worldwide infectious disease and osteoporosis threatens life prognosis by lumbar or hip fractures. Since more than half of the population infected with* H. pylori* lives in an aging society such as Japan, the relationship between* H. pylori *infection and osteoporosis is a very serious issue.

To investigate the factors associated with osteoporosis, we established the inclusion and exclusion criteria for the study subjects very carefully. At first, we defined the study subjects to be 50 years old or older because this age corresponds to an increase in risk of osteoporosis [15]. Premenopausal women were excluded because menopause affects bone metabolism strongly [16]. Furthermore, we excluded various diseases and drugs that are associated with bone metabolism as secondary osteoporosis. With adherence to these strict criteria, we were able to discern a relationship between* H. pylori *infection and osteoporosis.

In terms of the mechanism that* H. pylori* infection affects the onset of osteoporosis, the effects of local and systemic inflammatory response by* H. pylori *infection are considered very important [4].* H. pylori* infection causes chronic gastritis and induces an inflammatory response that may increase both the gastric and the systemic indexes of inflammatory cytokines [17], such as IL-1, 6, 8, and TNF-*α*, which may exert their effect in remote tissues [3, 9]. Since these cytokines are involved in the regulation of bone turnover,* H. pylori* infection may affect indirectly the bone turnover [4]. Moreover,* H. pylori* strains possessing CagA, which is a representative strong pathogenic factor related to atrophic gastritis, peptic ulcer disease, and stomach cancer [18, 19], are considered more virulent and capable of inducing increased local and systemic inflammatory responses [20]. However, there are regional differences in CagA strains. Approximately 100% of strains in Japan have CagA, whereas CagA is observed in only 30–40% of strains in Western countries [21]. Furthermore, there are two kinds of genetic polymorphism in CagA: the East Asian type and the non-East Asian type [22, 23]. Abe et al. reported that the grades of activity of gastritis and mucosal atrophy in East Asian type CagA were significantly more severe than that of non-East Asian type CagA [24]. Therefore, the East Asian type CagA strains may be more virulent than the non-East Asian type CagA strains. However, by international comparison of the CagA genetic polymorphism for* H. pylori*, the East Asian type CagA is not detected in Western countries, whereas most type of CagA are East Asian type in east Asia [25]. VacA and OipA genes are other pathogenic genes of* H. pylori*; however, most of these strains (VacA, OipA) are more strong pathogenic type in Japan compared with that of Western countries [26–28]. According to one report from Italy, Figura et al. reported that the prevalence of CagA-positive* H. pylori* infection was increased in patients with osteoporosis compared to controls [29], which is similar to our results in Japan.

Endoscopic gastric mucosal atrophy (EGA) in our data tended to correlate with osteoporosis. The decrease of dissolution of calcium salts by the decrease of gastric acid secretion in atrophic gastritis patients may also result in the malabsorption of calcium. The inflammatory response and gastric mucosal atrophy in Japanese patients with* H. pylori* infection are expected to be much more severe compared with that of patients in Western countries. Most of previous studies of the relationship between* H. pylori* infection and osteoporosis have reported no association [30–33]. However, these previous studies examined only 50–105 cases; multivariate analysis was not performed; and the reports were from Western countries only. The difference between Western countries and Japan in the influence of* H. pylori* infection on bone metabolism may be due to geographic difference of genetic polymorphisms of* H. pylori*.

In other words, the following factors were considered as the mechanism that* H. pylori* infection affects the onset of osteoporosis. At first the* H. pylori* infection to the gastric mucosa causes increase of inflammatory cytokines and mucosal atrophy in the stomach. Then, the increase of systemic inflammatory cytokines and severe gastric mucosal atrophy are caused by sustained infection of* H. pylori*. It is thought that the increase of systemic inflammatory cytokines induces differentiation of the osteoclast which is related to bone destruction. On the other hand, the decrease of gastric acid secretion by severe gastric mucosal atrophy causes a calcium malabsorption. Because the strong pathogenicity of* H. pylori* in Japan additionally exacerbates the increase of systemic inflammatory cytokines and calcium malabsorption, consequently osteoporosis may be caused.

Examination of the bone metabolism markers showed that BAP tended to be related to osteoporosis. Of osteoporosis patients, the mean age was considered young at 68.9 ± 7.3 years old, and 80.5% (33/41) of the cases were female, which suggests that most were a high turnover type of osteoporosis. Although NTX was not significantly related with osteoporosis, NTX activity may be influenced by the large circadian rhythm at the time of blood examination.

Our results demonstrated that PUD was risk factor of osteoporosis. Sawicki et al. reported that PUD is an independent risk factor for osteoporosis, although they did not investigate the* H. pylori* infection status [34]. In PUD patients, gastric and duodenal epithelium are defective, and inflammation at these sites may cause the malabsorption of calcium and other substances such as macroelements, which play a crucial role in mineral homeostasis and bone metabolism [35]. Additionally, dietary restrictions due to PUD may also have a negative influence on the dietary intake of calcium. On the other hand,* H. pylori* is the most important factor that causes PUD. In our study, 73.1% (19/26) (data not shown) of PUD patients were* H. pylori* positive, which suggests an influence of the* H. pylori* infection on the incidence of PUD.

We also investigated the relationship between osteoporosis and PPI use but did not observe a significant relationship with osteoporosis. PPI is a strong gastric acid secretion inhibitor and has been reported recently to correlate with bone fracture [36, 37]. However, most of these reports were retrospective studies. Furthermore, it has been reported that there are many patients with lumbar spine pressure fracture due to osteoporosis in PPI users and there are many patients with hiatal hernia and severe reflux esophagitis who require PPI as treatment [38].

Life style factors such as alcohol consumption and smoking were not related with osteoporosis in this study. In this study, approximately 80% of osteoporosis patients were women, and there were fewer drinkers and smokers in women than men. Therefore, it was suggested that nondrinkers and nonsmokers became the risk factor of osteoporosis in bivariate analysis. However, the nondrinkers and nonsmokers were finally regarded as confounding factor because the nondrinkers and nonsmokers were not risk factor in the multivariate analysis. According to one report, there is evidence of an effect on bone metabolism by smoking cessation [39]. It was suggested that it may be insufficient only in a smoking history, and the current smoking state may be important. Comorbidity, such as type 2 diabetes mellitus and hypertension, and LDA user were not related with osteoporosis in this study. It is known that type 1 diabetes mellitus is related to the decrease of bone mineral density (BMD). On the other hand, it is known that bone fracture risks are increased by type 2 diabetes mellitus; however, the BMD does not decrease [40]. In a study that included 3676 women, Cappuccio et al. reported that high blood pressure in elderly white women is associated with increased bone loss at the femoral neck [41]. Bauer et al. reported that although the fracture risk was similar among daily users of aspirin and nonusers, regular use of aspirin may have a modest beneficial effect on BMD in postmenopausal women [42]. While there were a small number of cases in this study, the examination of many cases is necessary to investigate these factors. Advanced age, female gender, and low BMI were associated with osteoporosis in this study, and these risk factors of osteoporosis were similar with the results of a previous report [43].

In conclusion, our results showed that* H. pylori* infection is a risk factor of osteoporosis in Japan. The relationship between* H. pylori *infection and osteoporosis is a crucial problem in Japan as an aging society with high prevalence of* H. pylori*. In cases of* H. pylori* positive females of 50 years or older (with peptic ulcer disease in particular), we should perform dual-energy X-ray absorptiometry (DXA) to prevent the risk of bone fracture.

## Figures and Tables

**Table 1:** Clinical characteristics (*n* = 200).

	Male	Female	*P* value	Total
	95 (47.5)∗	105 (52.5)∗		200 (100)∗
Patient profile				
Age (years)	62.8 (±7.7)∗∗	63.4 (±9.6)∗∗	0.637	63.1 (±8.8)∗∗
BMI (kg/m^2^)	23.6 (±2.6)∗∗	22.2 (±3.8)∗∗	0.002	22.9 (±3.4)∗∗
Alcohol consumption				
Nondrinker	36 (37.9)∗	86 (81.9)∗	<0.001	122 (61.0)∗
Drinker	59 (62.1)∗	19 (18.1)∗		78 (39.0)∗
Smoking				
Nonsmoker	44 (46.3)∗	94 (89.5)∗	<0.001	138 (69.0)∗
Smoker	51 (53.7)∗	11 (10.5)∗		62 (31.0)∗

*H*. *pylori* infection status				
*H*. *pylori* infection				
Negative	57 (60.0)∗	60 (57.1)∗	0.682	117 (58.5)∗
Positive	38 (40.0)∗	45 (42.9)∗		83 (41.5)∗

Comorbidity				
Diabetes mellitus (type 2)				
No	83 (87.4)∗	97 (92.4)∗	0.364	180 (90.0)∗
Yes	12 (12.6)∗	8 (7.6)∗		20 (10.0)∗
Hypertension				
No	61 (64.2)∗	81 (77.1)∗	0.044	142 (71.0)∗
Yes	34 (35.8)∗	24 (22.9)∗		58 (29.0)∗

Internal medical therapies				
Proton pump inhibitor (PPI)				
Nonuser	63 (66.3)∗	79 (75.2)∗	0.165	142 (71.0)∗
User	32 (33.7)∗	26 (24.8)∗		58 (29.0)∗
Low dose aspirin (LDA)				
Nonuser	82 (86.3)∗	96 (91.4)∗	0.267	178 (89.0)∗
User	13 (13.7)∗	9 (8.6)∗		22 (11.0)∗

Lumber DXA				
Bone mineral density (BMD) (g/cm^2^)	0.96 (±0.14)∗∗	0.81 (±0.16)∗∗	<0.001	0.88 (±0.17)∗∗
Young adult mean (YAM) (%)	91.1 (±13.5)∗∗	79.3 (±15.6)∗∗	<0.001	84.9 (±15.7)∗∗

Bone turnover marker				
Bone specific ALP (BAP) (U/L)	21.4 (±6.0)∗∗	23.4 (±6.6)∗∗	0.025	22.5 (±6.5)∗∗
Collagen type-I cross-linked N-telopeptide (NTX) (nmolBCE/L)	13.6 (±4.8)∗∗	14.8 (±5.2)∗∗	0.112	14.2 (±5.0)∗∗

Upper GI findings				
Reflux esophagitis (RE)				
No	89 (93.7)∗	102 (97.1)∗	0.313	191 (95.5)∗
Yes	6 (6.3)∗	3 (2.9)∗		9 (4.5)∗
LA-grade A	4	3		7
Grade B	2	0		2
Grade C	0	0		0
Grade D	0	0		0
Hiatal hernia				
No	47 (49.5)∗	62 (59.0)∗	0.175	109 (54.5)∗
Yes	48 (50.5)∗	43 (41.0)∗		91 (45.5)∗
Peptic ulcer disease (PUD)				
No	83 (87.4)∗	91 (86.7)∗	0.883	174 (87.0)∗
Yes	12 (12.6)∗	14 (13.3)∗		26 (13.0)∗
Gastric ulcer	5	10		15
Duodenal ulcer	4	2		6
Gastroduodenal ulcer	3	2		5
Endoscopic gastric mucosal atrophy (EGA)	1.9 (±1.8)∗∗	1.8 (±2.0)∗∗	0.811	1.8 (±1.9)∗∗
C-0	28	38		66
C-1	26	25		51
C-2	11	7		18
C-3	3	9		12
O-1	16	11		27
O-2	9	10		19
O-3	2	5		7

*Number (%), ∗∗median (±SD).

**Table 2:** Association between the likelihood of osteoporosis in bivariate and multivariate logistic regression analysis.

Covariates	Nonosteoporosis	Osteoporosis	Bivariate			Multivariate∗∗∗		
	159 (79.5%)∗	41 (20.5%)∗	Standardized coefficient	OR (95% CI)	*P* value	Standardized coefficient	OR (95% CI)	*P* value
Patient profile								
Age (years)	61.7 (±8.5)∗∗	68.9 (±7.3)∗∗	0.098	1.10 (1.06–1.15)	<0.001	0.124	1.13 (1.07–1.20)	<0.001
Sex								
Male	87 (54.7)∗	8 (19.5)∗		1.00 (reference)			1.00 (reference)	
Female	72 (45.3)∗	33 (80.5)∗	1.606	4.98 (2.17–11.47)	<0.001	1.561	4.77 (1.78–12.77)	0.002
BMI (kg/m^2^)	23.4 (±3.3)∗∗	21.0 (±3.1)∗∗	−0.244	0.78 (0.69–0.89)	<0.001	−0.238	0.79 (0.68–0.92)	0.002
Alcohol consumption								
Nondrinker	91 (57.2)∗	30 (74.2)∗		1.00 (reference)			1.00 (reference)	
Drinker	68 (42.8)∗	11 (26.8)∗	−0.841	0.43 (0.20–0.94)	0.035	0.163	1.18 (0.39–3.58)	0.774
Smoking								
Nonsmoker	102 (64.2)∗	36 (87.8)∗		1.00 (reference)			1.00 (reference)	
Smoker	57 (35.8)∗	5 (12.2)∗	−1.392	0.25 (0.09–0.67)	0.006	−0.198	0.82 (0.20–3.31)	0.781

*H*. *pylori* infection status								
*H*. *pylori* infection								
Negative	102 (64.2)∗	16 (39.0)∗		1.00 (reference)			1.00 (reference)	
Positive	57 (35.8)∗	25 (61.0)∗	1.132	3.10 (1.52–6.33)	0.002	1.674	5.33 (1.73–16.42)	0.004

Comorbidity								
Diabetes mellitus (type 2)								
No	141 (88.7)∗	39 (95.1)∗		1.00 (reference)				
Yes	18 (11.3)∗	2 (4.9)∗	−0.912	0.40 (0.09–1.81)	0.234			
Hypertension								
No	111 (69.8)∗	31 (75.6)∗		1.00 (reference)				
Yes	48 (30.2)∗	10 (24.4)∗	−0.293	0.75 (0.34–1.64)	0.467			

Internal medical therapies								
Proton pump inhibitor (PPI)								
Nonuser	114 (71.7)∗	28 (68.3)∗		1.00 (reference)				
User	45 (28.3)∗	13 (31.7)∗	0.162	1.18 (0.56–2.47)	0.669			
Low dose aspirin (LDA)								
Nonuser	141 (88.7)∗	37 (90.2)∗		1.00 (reference)				
User	18 (11.3)∗	4 (9.8)∗	−0.166	0.85 (0.27–2.65)	0.775			

Lumber DXA								
Bone mineral density (BMD) (g/cm^2^)	0.94 (±0.13)∗∗	0.65 (±0.07)∗∗						
Young adult mean (YAM) (%)	90.4 (±12.3)∗∗	63.4 (±6.5)∗∗						

Bone turnover marker								
Bone specific ALP (BAP) (U/L)	22.0 (±6.5)∗∗	24.4 (±5.7)∗∗	0.055	1.06 (1.00–1.11)	0.038	0.045	1.05 (0.98–1.12)	0.184
Collagen type I cross-linked N telopeptide (NTX) (nmolBCE/L)	14.0 (±4.9)∗∗	15.0 (±5.6)∗∗	0.037	1.04 (0.97–1.11)	0.258			

Upper GI findings								
Reflux Esophagitis (RE)								
No	151 (95.0)∗	40 (97.6)∗		1.00 (reference)				
Yes	8 (5.0)∗	1 (2.4)∗	−0.751	0.47 (0.06–3.88)	0.485			
LA-grade A	6	1						
Grade B	2	0						
Grade C	0	0						
Grade D	0	0						
Hiatal hernia								
No	83 (52.2)∗	26 (63.4)∗		1.00 (reference)				
Yes	76 (47.8)∗	15 (36.6)∗	−0.462	0.63 (0.31–1.28)	0.201			
Peptic ulcer disease (PUD)								
No	144 (90.6)∗	30 (73.2)∗		1.00 (reference)			1.00 (reference)	
Yes	15 (9.4)∗	11 (26.8)∗	1.258	3.52 (1.47–8.42)	0.005	1.606	4.98 (1.51–16.45)	0.008
Endoscopic gastric mucosal atrophy (EGA)	1.7 (±1.8)∗∗	2.4 (±2.2)∗∗	0.185	1.20 (1.01–1.43)	0.039	−0.228	0.80 (0.60–1.05)	0.106
C-0	53	13						
C-1	46	5						
C-2	14	4						
C-3	8	4						
O-1	21	6						
O-2	14	5						
O-3	3	4						

∗Number (%).

∗∗Median (±SD).

∗∗∗Models adjusted for age, sex, BMI, alcohol consumption, smoking, *H*. *pylori* infection, BAP, PUD, and EGA.

Model square test: *P* < 0.01.

Nagelkerke *R* ^2^ = 0.454.

Hosmer-Lemeshow goodness of fit test: *P* = 0.900.

Discriminant hitting ratio = 86.0%.

## Abbreviations

OR: Odds ratio
CI: Confidence interval
SPSS: Statistical packages for social sciences.
